# Chronically Implanted Pressure Sensors: Challenges and State of the Field

**DOI:** 10.3390/s141120620

**Published:** 2014-10-31

**Authors:** Lawrence Yu, Brian J. Kim, Ellis Meng

**Affiliations:** 1 Department of Biomedical Engineering, University of Southern California, 1042 Downey Way, DRB-140, Los Angeles, CA 90089-1111, USA; E-Mails: lawrence.yu@usc.edu (L.Y.); brianjk@usc.edu (B.J.K.); 2 Ming Hsieh Department of Electrical Engineering, University of Southern California, 3740 McClintock Ave, EEB-100, Los Angeles, CA 90089-2560, USA

**Keywords:** implantable pressure sensors, implantable pressure transducers, biocompatibility, telemetry, drift

## Abstract

Several conditions and diseases are linked to the elevation or depression of internal pressures from a healthy, normal range, motivating the need for chronic implantable pressure sensors. A simple implantable pressure transduction system consists of a pressure-sensing element with a method to transmit the data to an external unit. The biological environment presents a host of engineering issues that must be considered for long term monitoring. Therefore, the design of such systems must carefully consider interactions between the implanted system and the body, including biocompatibility, surgical placement, and patient comfort. Here we review research developments on implantable sensors for chronic pressure monitoring within the body, focusing on general design requirements for implantable pressure sensors as well as specifications for different medical applications. We also discuss recent efforts to address biocompatibility, efficient telemetry, and drift management, and explore emerging trends.

## 1. Introduction

Pressure in various organs of the body (e.g., brain, eye, heart, bladder) is highly regulated and its value can provide an indication of patient health or disease progression. In diseases where the ability to regulate these pressures is lost, impairment of function or death may result; even at the microscopic level, cell damage may be induced in the presence of abnormal pressure. Monitoring the absolute value of and variation of internal pressures allows diagnosis and tracking of the progress of medical intervention.

In current medical practice, it is common to obtain a single patient pressure data point in the clinic. However, this information only provides a limited snapshot of the dynamic pressure profile within the organ of interest and does not capture peak or trough values or changes in the profile which may provide important clues to inform appropriate intervention strategies [1,2]. Also, measurements obtained may be perturbed by the stress experienced by some patients when visiting clinical settings [3]. Therefore advances in sensing technology to enable convenient, accurate, and continuous pressure monitoring that can extend to settings outside the clinic may enable more effective disease management.

Noninvasive methods for measuring pressure have been explored extensively. These methods infer pressure as tissues interact with high-energy waves imposed on the body in the form of sound or electromagnetic radiation (e.g., light, X-ray). However, these methods do not provide adequate precision and accuracy [4]. Catheterization enables targeted probing of pressure in specific body regions, and this invasive method grants the accuracy needed for effective chronic monitoring, currently serving as the gold standard for patient care in many conditions. However, prolonged catheterization of bed bound patients hinders normal, ambulatory behavior (e.g., psychological white-coat effects that shows false positives) and the catheter breach across the skin leaves the patient prone to infection [2,5–7].

Fully implantable sensors provide targeted measurement of pressure in the body without the infection risks posed by catheters or wires. Devices that integrate a sensing element with telemetry, archaically named endoradiosondes or transensors, have been in development for over half a century [8–10]. Such devices enable a clinician to easily monitor the patient over the lifetime of the implanted sensor. The rapid improvement of microfabrication technologies has enabled the production of low cost, highly accurate sensors that may be safely implanted into patients for chronic monitoring. Over the last few decades, there has been steady progress in the development of technologies to achieve clinically relevant implantable pressure sensors. The reader is referred to past reviews on pressure sensor types for biomedical applications for more comprehensive coverage of related developments [11–13]. In this paper, we focus the discussion on implantable pressure sensors for long-term pressure monitoring within the body, including their requirements and specific considerations for different pressure sensing applications. Finally, we highlight recent work and trends in chronic pressure sensing development.

### 1.1. Challenges for Implantable Pressure Sensors

The *in vivo* environment presents a host of engineering challenges that must be considered in the development of implantable pressure sensors. The sensing element must be able to transduce pressure over the range of clinically normal and abnormal values with sufficient resolution to inform treatment. A telemetry system is required to transmit the data to an external reader and may also serve to power the implanted system. For achieving both, considerations of the interactions between the implanted system and the body, including biocompatibility, surgical placement, and patient comfort are essential to achieve a feasible design. Key requirements for chronic implantable pressure sensors are discussed.

#### 1.1.1. Calibration

The prevailing method of pressure measurement for *in vivo* applications is differential (gauge) in which atmospheric pressure is designated as the baseline or “zero” for the measurement. This reference pressure value is not only dependent on the atmospheric temperature but a variety of other factors such as temperature and even time of day; these variations give rise to large baseline (or zero) drift. Another type of differential pressure measurement is taken as the difference in pressure taken at two points, where an example might be the pressure from outside the bladder subtracted from the pressure inside [14]. Absolute pressure measurement is the measurement of pressure difference compared to a perfect vacuum. The challenge of creating an absolute pressure sensor partially stems from the challenge of creating a perfect vacuum and ensuring that it remains relatively stable over an extended period of time [15]. Currently, all *in vivo* pressure measurements are reported as gauge, so a calibration (“zeroing”) procedure must be performed prior to implantation in order to report a gauge measurement with an absolute pressure transducer.

#### 1.1.2. Integration with the Body

For chronically implanted sensors, the interaction with the body's warm, saline environment can be a cause for concern especially for electrically active circuits and systems which are vulnerable to ionic contamination [16,17]. The electrolyte rich aqueous environment within the body is conductive and can cause inadvertent shorts or electrical leakage if electronic circuits are exposed. Proper hermetic encapsulation is required to protect the electronics from water intrusion that can result in sensor drift and device failure. Furthermore, ingress of gas (e.g., oxygen) can cause oxidation of interconnect metals such as solders, which in turn can lead to attachment failure [18]. The materials incorporated should also be resistant to corrosion [17].

Implantable sensors must also manage the immune response occurring at its surfaces. The pressure sensing function may be adversely affected by the body's action to isolate and expel any foreign bodies [19]. Potential mitigation strategies include the selection of biocompatible materials that have a track record of use in FDA-approved implants or designs that minimize tissue irritation [16]. Any material or construction strategy will need to be evaluated in biocompatibility studies on the final device. Also, the achievable performance in the presence of the inevitable fibrous tissue encapsulation or other cellular or blood-based biofouling needs to be carefully evaluated, especially if the device operates in a mechanical mode.

In addition to the foreign body response of the body to the implanted device, the sensor must be carefully designed to minimize any damage to tissues during implantation and due to its chronic presence. Such disruptions may exacerbate the normal foreign body response and further worsen the chronic *in vivo* performance of the implant. Designs that anticipate and mitigate the immune response can improve sensor integration and preserve long-term performance.

#### 1.1.3. Telemetry

Wireless telemetry can be used to provide power to and/or receive pressure data from the body. This is commonly accomplished using radio frequency (RF)-based antennae or inductively coupled transmission [20]. Telemetry may take on one of two forms for implantable sensors: (1) active telemetry, where the implanted system contains active electronics (e.g., amplifiers, microcontrollers), for which a power source is necessary, either through an onboard battery or transmitted power signal; or (2) passive telemetry, where the implanted system is completely passive (no power necessary) and is usually interrogated externally as load changes to an external coil (e.g., resonant frequency shifts of LC tanks). For the active case, long term monitoring requires long lasting power sources, which may be rechargeable. However, implantable power sources are still large in size. This disadvantage may be overlooked in the case where the enabled signal processing can achieve improved signal stability in the long term. Passive devices may be preferred for advantages in simplicity of circuitry on the implant size and smaller implant footprint. In either case, issues related to communication over distances persist; RF signals quickly dissipate in soft tissue and bone while inductive coupling requires proper placement and alignment of internal and external coils for proper signal transfer. A large body of work exists that describes methods to improve the coupling efficiency between the external coil and implanted device, regardless of its position [21–31].

#### 1.1.4. Drift and Long-Term Accuracy

To achieve reliable long-term measurements, implanted sensors should possess a stable, consistent response over their lifetime. Shifts in response should only be due to pressure and not other interfering factors. Signal drift encountered in sensors can be divided into two categories: (1) offset drift, where the baseline measurement slowly drifts to obscure the desired pressure measurement and (2) sensitivity drift, where the devices face slow reductions in sensitivity over time [32]. The causes of drift are attributed to changes in the sensor performance independent of the environment (*i.e.*, material aging and mechanical fatigue) and changes in the environment in which the device is implanted. The *in vivo* environment is dynamic; pressure is just one of many parameters that change over time. Assuming that a device incorporates appropriate material choices and sensing operational principles geared towards implantable applications, a drift and poor long-term accuracy stem largely from interactions with the wet, implanted environment such as unavoidable tissue encapsulation. For this reason, it is crucial for implanted sensors to have drift compensation circuits or zeroing functions that can allow for correction to provide reliable measurements over the period of implantation.

## 2. Pressure Measurement Principles

A brief overview of common pressure sensing modalities suitable for implantable systems is presented here.

### 2.1. Sensing Methods

#### 2.1.1. Membrane-Based Sensors

An overwhelming majority of implantable pressure sensors utilize a simple design consisting of a membrane and a sealed cavity; the membrane element responds to and deflects under pressure [8–10,33–45]. Because of the simplicity of the design, geometry and materials have been adapted for a wide variety of applications in addition to *in vivo* environments [46–50]. Furthermore, piezoelectric or capacitive sensing of membrane deflection can be integrated easily with telemetry electronics.

**Figure 1.**
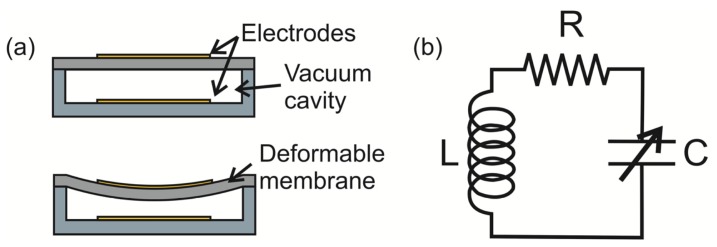
(**a**) Schematic of operation of a capacitive-based membrane pressure sensor. Diaphragm deflects under pressure, changing the effective distance between two parallel plates, and thus increases the measured capacitance across the plates; (**b**) Circuit model of passive LC tank, commonly used for inductively coupled telemetry of capacitive-based sensors.

The miniaturization advantages of microelectromechanical systems (MEMS) technology can scale these deformable membranes into a small form factor and create complete micro-scale systems that can easily be implanted within the body. MEMS-based membrane pressure sensors most commonly use either capacitive or piezoresistive methods to transduce the membrane deflection into an electrical signal. In the case of capacitive sensing, an electrode is placed on the top (deformable) and bottom (rigid) surfaces of the sealed cavity. Deflection of the membrane causes changes in the capacitance measured between the electrode pair (Figure 1a). For piezoresistive sensing, a piezoresistor is patterned onto the membrane surface, and deflection of the membrane is transduced into a change in resistance, usually measured via a bridge circuit.

**Figure 2.**
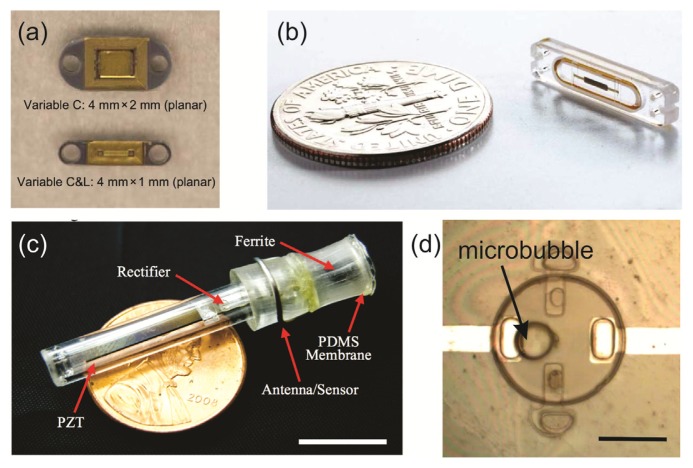
Examples of implantable pressure sensor systems currently in development: (**a**) A capacitive-based sensor for measurements of intraocular pressure [39]; (**b**) Capacitive-based sensor developed by CardioMEMS for measurements of blood pressure; (**c**) Inductor-based sensor with piezoelectric energy harvester for measurements of bladder pressure (scale bar 10 mm) [45]; (**d**) Micro-bubble based pressure sensor for measurements of intracranial and bladder pressure (scale bar 100 μm) [61].

The selection of one method over the other is dictated by the collective combination of advantages and disadvantages. Capacitive sensing allows for high pressure sensitivity and low temperature drift compared to piezoresistive, but suffers from the need for on-site detection/amplification circuitry due to small active capacitances [42,51]. Arraying capacitive sensors can improve sensitivity [52,53]. In piezoresistive-based membrane pressure sensors, the amplifier circuitry can be placed on the external circuit, but suffers from low-pressure sensitivity and high temperature drift, requiring temperature compensation. The use of a passive LC resonating circuit, or LC tank (Figure 1b), allows for simple wireless transmission of capacitive changes induced by pressure compared to wireless strategies for piezoresistive sensors; this allows reduction in implant size and simplifies the required sensing circuitry [54,55] (Figure 2a,b).

#### 2.1.2. Alternate Transduction Schemes

Other transduction schemes have also been investigated to address the challenges of pressure sensors outlined above. To improve biocompatibility and tissue integration, alternative transduction schemes that do not require hermetic sealing and utilize biocompatible polymers have been investigated, such as the use of localized gas bubbles that can be harnessed for pressure transduction [56] (Figure 2d). Alternatives to telemetry have been explored as well, such as the implementation of a microfabricated spiral bourdon tube [57] and manometer that can be interrogated via visual inspection or through the use of X-ray imaging [38,58]. It is also interesting to note that the nervous system utilizes strain sensitive cells called baroreceptors; these have inspired the development of biomimetic strain sensors for applications in pressure transduction [59,60].

### 2.2. In Vivo Pressure Sensor Requirements

For chronic implanted sensors, the design is guided by a number of requirements beyond the initial selection of a pressure transduction scheme. Select requirements are reviewed and discussed.

#### 2.2.1. Size

The size of the implant is application dependent and should be sufficiently miniaturized so as to allow appropriate sensor placement in the body (*i.e.*, compatible with implantation techniques and anatomical restrictions) and integration with telemetry components (which further increase the footprint). By leveraging advances in micromachining technologies, MEMS-based sensors have been developed that minimize the overall footprint and mass. The overall system size can be further reduced by combining sensors with application specific integrated circuits (ASICs) instead of discrete electronic components. However, inductive powering coils and batteries are still relatively large and impose limits to miniaturization.

#### 2.2.2. Range and Precision

**Figure 3.**
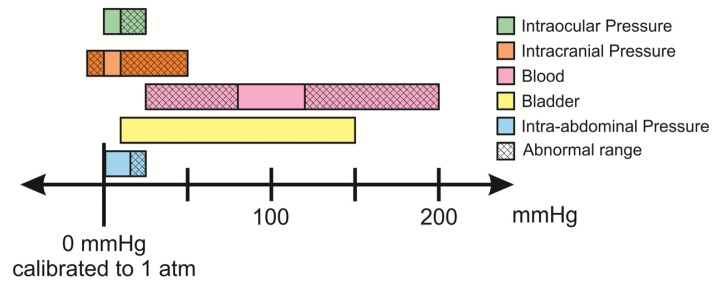
Relevant pressure ranges for *in vivo* pressure monitoring for diagnostic applications [62–67].

A number of pressures within the body are relevant indicators in various conditions and diseases. Taken together, these pressures span the range of −10 to 200 mmHg when taken in reference to atmospheric pressure. Pressure ranges of interest, including abnormal pressure ranges, for in different areas of the body are listed in Table 1 and graphically presented in Figure 3. Sensors should be able to measure both clinically relevant normal and abnormal pressure ranges. A commonly accepted specification for sensor precision across the majority of the *in vivo* applications mentioned in Table 1 is a measurement with deviation of ±1 mmHg, or 5%–10% of the clinically normal range.

#### 2.2.3. Calibration

Ideally, the measured baseline pressure measurement should remain constant under constant pressure conditions. In practice, this value can drift significantly over the lifetime of the implanted sensor. The baseline drift in the commonly used gauge pressure measurement may be attributed to temperature, biofouling, and even static discharge effects [68–70]. As an example, the observed range for baseline drift for a commonly used commercial sensor was ±5 mmHg, or up to 30% of the measurement range for certain clinical applications [71,72]. Paradoxically, this is at odds with requirements for precision, and limits the utility of long-term recordings of implanted pressure sensors [5,73–75]. This issue requires further attention to promote clinical adoption of implanted transducers.

#### 2.2.4. Frequency Response

**Figure 4.**
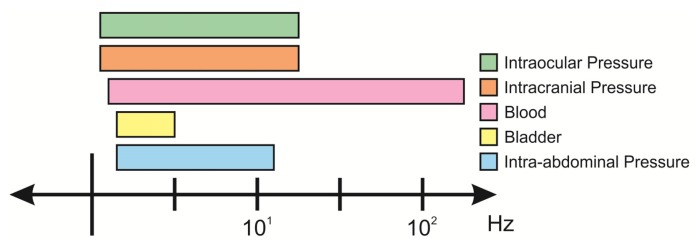
Relevant frequency bandwidths for varying pressure signals *in vivo* [23,32,82–85].

Nearly all fluid systems in the body are dynamic; fluids are constantly generated and drained, both actions that contribute to the pressure profile over time. Pressure transducers should be able to resolve and distinguish normal and abnormal physiological hydrodynamics. The dynamics of internal pressures in the body vary and the frequency response of the sensor should therefore be selected accordingly (Figure 4). A highly responsive sensor will enable data acquisition and Fourier analysis of waveforms, where flow and other useful biometrics may be inferred [76–78]. Sensors with poor frequency response and low slew rates will not properly capture transient changes in pressure, which can be critical indications of disease state [78–81].

#### 2.2.5. Materials and Packaging Considerations

In considering materials and packaging methods for implantable pressure sensors, several points must be considered: (1) acceptable tissue integration (*i.e.*, low cytotoxicity); (2) hermetic encapsulation for protection of active circuits and (3) long-term mechanical stability in the *in vivo* environment. Due to the inevitable foreign body response against the implanted sensor, device materials must be chosen to try and limit biofouling for more reliable, long-term performance of the device [16]. Sterilization is also an important factor to consider when choosing materials; devices should be able to withstand autoclave sterilization heat or chemical sterilization processes (e.g., ethylene oxide or plasma) [86,87]. Further, additional considerations for device design (e.g., removing sharp corners) can also benefit integration [88]. Efforts to encapsulate devices in drug-based coatings (e.g., anti-inflammatory) have also shown to limit the immune response [19,89]. For sensors with active circuitry, proper hermetic packaging is necessary to impede electrolyte intrusion to prevent device failure and sensor drift. For membrane based transducers that rely on the mechanical stability of the membrane material, material selection must be carefully considered for long-term operation [17,90].

#### 2.2.6. Telemetry and Circuitry

The design of electronics for implant telemetry requires careful management of a number of tradeoffs that affect power consumption, antenna size, and transmission frequency. Electromagnetic radiation characteristics must carefully prioritize safety and performance while addressing the challenges of power and data transmission in the *in vivo* environment. Standards have been set that limit tissue heating and radiation specific absorption rate (SAR), [91,92]. A licensed frequency band for communications, medical implants communications service (MICS), encourages development of telemetry systems within 402–405 MHz, but newer studies suggest operating at higher frequency (2+ GHz) for optimum performance [21]. Dielectric heating during MRI examinations should be considered (especially if the implant is situated in a critical location in the body), so analysis of MR heating should be done prior to implantation [93]. Another area of concern for implants would be interactions during X-ray examination, which can damage microcontroller and non-volatile storage.

## 3. Application Specific Requirements

In addition to the requirements listed above, there are several criteria that are application specific. The etiology and current gold standard of measurement is detailed in the following sections for the most common clinical pressure sensing applications. Critical parameters for proper pressure sensor operation within the reviewed applications are summarized in the following table:

### 3.1. Intracranial Pressure

Intracranial hypertension is an acute and chronic condition with a variety of causes that include traumatic brain injury, aneurysms, brain tumors, hydrocephalus, stroke, and meningitis. Standard treatment is the relief of fluid pressure through a lumbar puncture (spinal tap) or external ventricular drain (EVD) [78], where an opening in the skull is created and cerebrospinal fluid (CSF) is drained. For hydrocephalic patients, fluid pressure is shunted, typically to the abdominal cavity, with a surgically implanted catheter. Unfortunately, shunt failures are common, highlighting the importance of monitoring and regulation of CSF pressure for treatment and recovery [66,97–99]. Most importantly, long-term monitoring of ICP provides a quantitative method for diagnosis in contrast to vague symptoms (e.g., headaches, nausea). Two commonly used EVDs are the Codman Microsensor ICP (Codman & Shurtleff, Inc., Raynham, MA, USA) and Camino (Integra LifeSciences Corporation, Plainsboro, NJ, USA), which feature a sensor-tipped catheter that enables location-specific pressure measurement in the intracranial space [100]. Unfortunately, long-term monitoring with the EVD confines patients to the clinic and the extended period of opening of the blood-brain barrier increases risk of meningitis and other infections (exacerbating intracranial hypertension) [4].

Implantable telemetric pressure sensors promise improved precision of measurements without the disadvantages current technologies. However, aggressive immune response within the blood brain barrier can lead to significant biofouling and drift in sensor response [10,74,101]. Robust sensor design should seek to minimize immune response and drift to be effective for monitoring of intracranial hypertension.

### 3.2. Intraocular Pressure

The monitoring of intraocular pressure (IOP) is crucial for the diagnosis and monitoring of glaucoma. The Goldmann tonometer is the prevailing standard used to quickly and noninvasively infer IOP for diagnosis of glaucoma, but unfortunately this only provides a snapshot of the dynamically changing intraocular pressure and is of limited use for monitoring of disease progression [2,13,95]. Implantable sensors enable the clinician to accurately monitor IOP for patient response to treatment (e.g., assessing efficacy of surgically implanted drainage devices during the course of recovery). Fortunately, a multitude of treatment options exist for glaucoma, and the addition of long-term IOP information would help clinicians more accurately tailor the treatment program for the patient.

The design of implantable sensors must take into account the frequent movement of the eye, as implant placement may damage peripheral tissue and motion artifacts can obscure measurement [13]. The sensor should be placed so as not to block the visual path. Sensor design can take advantage of optically clear tissue that allows for visual interrogation, which obviates the use of complicated electronics for telemetry for the construction of a completely passive sensor. Active sensors may also utilize photovoltaics for long-term powering within this environment. As such, the development of IOP sensors has resulted in interesting designs that exploit the advantages of the optically clear environment.

### 3.3. Cardiovascular Pressure

Chronic blood pressure monitoring is critically needed to monitor various conditions of the cardiovascular system (e.g., restenosis, hypertension, and heart failure) as well as to assess the efficacy of surgical interventions as is the case in monitoring repaired aneurysms [102]. Though the long term efficacy of blood pressure monitoring can be justified, the current gold standards of blood pressure measurement, namely external pressure cuffs or intra-arterial catheter-based systems fall short due to lack of patient comfort, infrequent measurement, possible occlusion of blood flow, and long-term complications such as trauma and infection. As these approaches require the patient to be tethered to the measurement system in a clinical setting, inaccuracies can be introduced as was observed by a “white coat hypertension” due to psychological effects [103]. The long-term monitoring of blood pressure can be greatly improved using implantable blood pressure sensors that allow for continual monitoring without hindering daily activities.

Two industry pressure sensor developments of note are the Remon ImPressure/RemonCHF from Remon Medical Technology (acquired by Boston Scientific, Natwick, MA, USA) for measurement of pressures within an aneurysm sac following endovascular aneurysm repair (EVAR) [104] as well as the first FDA approved implantable blood pressure device, the EndoSensor/CardioMEMS HF System from CardioMEMS (recently acquired by St. Jude Medical, Saint Paul, MN, USA) for detection of heart failure within the pulmonary artery. Given the prevalence of heart disease, large strides have been made in implantable cardiovascular pressure sensors, however there is still room for improvement and technology advances.

### 3.4. Bladder Pressure

Urinary incontinence is a common issue within the United States (affecting 13 million adults [105]) currently diagnosed using a series of tests collectively known as urodynamics. Among one of these tests is a cystometry study, where a catheter-based pressure sensor is inserted intraurethrally to access the bladder and assess pressure variations. Unfortunately, though this is the current gold standard, this catheterization process is both painful and non-physiological, which can compromise the accuracy of the test results. The fact that these tests are also conducted within the doctor's office in a 20–40 min span can provide unreliable results and underdiagnose various conditions as pressure variations may be obscured by psychological white-coat effects and short measurement times [106,107]. In addition, measurements are plagued by catheterization, such as disturbances along the catheter line and different resonance and response time delays due to fluid inertia. For chronic monitoring, these catheter-type devices are not viable as they are prone to infection and stone formation within the bladder [108].

Another effort that can benefit from implantable pressure sensors within the bladder are devices for neuromodulation, where patients can undergo electrical stimulation-based rehabilitation to either inhibit or promote bladder activity. Chronic bladder pressure measurements can enable closed loop interventions to provide more efficient and reliable treatment. The development of discrete wireless implantable sensors for the bladder allows for the necessary chronic monitoring of the pressures within the bladder, while maintaining the patient's quality of life.

### 3.5. Intra-Abdominal Pressure

Abnormalities in pressure within the thoracic cavity have multifactorial etiologies (e.g., edema, pneumothorax, sleep apnea) and benefits greatly from long-term monitoring [77]. Current methods utilize measurements of stomach distension or suprasternal placement of strain sensors [109] to infer pressures within the thoracic cavity, but these noninvasive approaches lack accuracy and specificity, limiting clinical use. Implanted pressure sensors must be able to withstand significant mechanical displacement of organs in the thoracic cavity during respiration.

## 4. Sensing Technologies and State of the Field

The focus of this following discussion will be the current state of the field in the development of improved system packaging, telemetry methods, and drift compensation for implantable pressure sensors.

### 4.1. Material Selection and Hermetic Packaging

A wide range of materials including metals (e.g., nitinol, titanium, platinum), polymers (e.g., liquid crystal polymers [31], Parylene, silicone rubber), and various inorganic (carbon nanotubes [15]) and ceramics (e.g., alumina) exhibit low cytotoxicity and sufficient immuno-inertness within the body and are therefore potential candidates for selection as biocompatible materials for sensor construction [16]. However, many MEMS sensors rely on the bulk micromachining of silicon and the use of non-biocompatible photoresists and epoxies such as SU-8 [31]. If the use of these materials cannot be avoided, it is necessary to protect the body from these materials using a hermetic packaging system. Caution must be exercised in the selection of biocompatible polymers for pressure sensing membranes, such as silicone [43] or liquid crystal polymers [55]. These polymers have been found to have significant water absorption once implanted within the body (or in benchtop soak tests) that have caused significant drift or leaks within their sealed cavities.

Beyond the materials used for the sensing element and circuit system, biocompatible materials for interconnects are also in development as crucial parts of the system. Typically lead free, gold-tin solders have been used [31], along with conductive epoxies that are encapsulated to prevent leaching into the body. Hermetic packaging can prevent failure of implanted active circuitry and conversely, prevent toxic materials from circuitry from reaching the surrounding biotic environment via leaching. A common approach to hermetic packaging uses a sealed metal casing [14,63,89] that houses the sensing and circuitry components of the system, or other material systems such as anodically bonded silicon-to-glass [41,110], sintered and fused ceramic enclosures [55], or hermetic welding [110]. For some membrane-based pressure sensors, the hermetic packaging can interfere with proper pressure transduction to the pressure sensitive membrane. In these cases, a window within the casing is created, typically with its own membrane that allows for deflections with pressure. The pressure is then conducted to the sensing membrane of the pressure sensor using an incompressible fluid (e.g., silicone gel or oil) that allows for low attenuation transfer of the pressure from the external package to the actual sensor [25,111].

To provide additional hermeticity and improved biocompatibility to a system, a secondary encapsulation typically with a silicone rubber or a Parylene coating is applied [19,39,93,112–114]. These coatings were found to increase the biocompatibility and reduce offset drift of these implantable pressure sensors. However, the conformality of the coating must be carefully monitored, as silicone rubber may have poor coverage and reveal cytotoxic regions at sharp edges of the device [115]. The addition of a silicone or Parylene coating over the membrane structure was found to reduce sensitivity [39,57,93,112,116], and thus degrading the signal-to-noise ratio and compromising the measurement resolution. Furthermore, PDMS encapsulation was found to negatively impact frequency response, causing a 1–2 s delay in the pressure measurement due to increased viscoelastic nature of the membrane [117]. Epoxies have also been used to encapsulate circuitry; some must be stained dark in order to reduce light incidence on ASICs [89] that may affect their performance.

Implantable pressure systems may take advantage of drug coatings to reduce the adsorption of proteins and other cellular materials (e.g., blood) on the membrane that could cause poor sensor performance. Antithrombogenic or heparin coatings for their devices allowed for reduced clotting for sensors implanted within arteries [89]. In another effort, sodium 2-acrylamido-2-methylpropyl sulfonate (AMPS) coating was adhered to the silicone encapsulation layer of their device to prevent the deposition of salts on their deflecting membrane to maintain consistent sensor operation [19].

Ideally, the pressure sensor format should minimize the need for bulky, hermetic packaging and utilize a material set that appropriately balances sensing and biocompatibility requirements. The micro-bubble transducer sets out to achieve this goal by eliminating the hermetic packaging around the sensing element (bubble) and utilizing a transduction mechanism (electrochemical impedance) that leverages the wet environment. The exclusive use of Parylene C and thin-film platinum reduces the complexities of multi-material implantable system that necessitates biocompatible encapsulation [61].

### 4.2. Telemetry

An in-depth review of various active and passive telemetry methods for implanted sensors is beyond of the scope of the present work, for a further information the reader is directed to the following publication [20]. Both active and passive telemetry methods have been explored that allow for implanted pressure sensors to transmit sensor data outside of the body for monitoring or to allow external units to send in power signals for active circuitry. In looking at the challenges associated with telemetry for chronically implanted pressure sensors, two issues arise, namely: (1) powering methods of the implanted sensor and system for continual use in the long term and (2) improving the coupling between the implanted and external coils as well as reducing the attenuation between them for more efficient power and data transfer. Approaches within literature specific to implanted pressure sensors that address these two issues will be presented within this section.

Currently, to transmit power to application specific integrated circuitry (ASICs) that allow for signal processing within the implanted device, two main methods are employed: battery-based approaches that have a limited lifetime, and RF based approaches that require coupling efficiency. For devices intended for acute monitoring (e.g., 1 week [19]) or for applications where the data transmission frequency is selected to maximize range instead of requiring a certain frequency for power transmission [14], a battery-based system can be ideal. To minimize power consumption and extend battery life, one approach is to use circuitry that turns the system on for 10 s every 5 min, in essence creating a “sleeping mode” for the sensor to conserve battery life [118]. However, these methods do not provide truly continuous monitoring of pressure and are appropriate when occasional sampling is sufficient.

For longer implant durations (months to years), an RF-based inductive coupling approach is normally used to transmit power signals between implanted and external coils. The coupling efficiency between the implanted and external transmitter/receivers is of concern, as the RF signal needs to be continuously transmitted in order for the sensor to operate in a continuous mode. Also, an external bulky transmitter must be continuously worn. In an effort to reduce the bulky equipment required for continuously monitoring systems to improve patient quality of life, a hybrid system was created that combined the battery and RF charging components [26,27,83]. This implantable RF-based rechargeable battery design allows for the sensor to operate and transmit signals using a battery during the day eliminating the need for an externally worn transmitter, and then recharge the battery at night using an external coil within the bed of the patient. Because of the reliance of battery operation during normal measurements during the day, specific methods to increase the lifetime of the lithium battery were implemented, such as the recharging of the battery to under the maximum power, such that the number of recharge cycles was extended. Beyond that, the battery itself has the capacity to run the implanted system for 48 h, but is recharged on a 24-h basis. A hybrid system such as this would be able to eliminate the need for a continuous power signal and mandatory wearable external transmitter and provide a more efficient strategy for long-term continuous pressure monitoring.

Implantable sensors for small animals or patients in an ambulatory setting can also have issues with improper coupling between the implanted and external coils leading to poor power or data transmission. Current efforts set to improve two aspects of transmission by either improving or circumventing coupling issues between the two coils or also by reducing the attenuation-related effects using novel coil designs. In the case of the former, there have been efforts in the development of adaptive power control technology [25,30] that can enable the sensing mode if the power level is high enough to run the sensor and onboard ASIC system, or can store the transferred power to a capacitor if the power received is not high enough. This technique circumvents poor coupling and uses smart implant technologies to operate when the power becomes sufficient. In other efforts, one system utilizes three external coils with a system that allows for the external coil that couples the best with the implanted coil to switch on, while the others remain off [23]. This leads to short down time of the system while the correct coil is being chosen, but can ensure proper coupling regardless of the position of the implanted device. As mentioned previously, RF-battery hybrid systems eliminate the need for continuous RF coupling and can use a large external coils (within the bed of the patient, recharging of the device occurs at night) that would reduce coupling factor variation.

To reduce attenuation, current devices set to improve the dielectric mismatch for implanted devices or improve the quality factor of their transmitter coils. For example, certain LC tank-based systems face issues once implanted due to changes in the dielectric constant of the surrounding wet environment compared to air (ε_r_ ∼ 1 in air and ε_r_ ∼ 80 for saline) [55]. These changes in the dielectric constant of the surrounding environment increases the total capacitance of the system and in essence decreases the resonant frequency as well as the quality factor of the system [55]. The silicone encapsulation however, though it has been shown to causes changes to the sensitivity of the device, helps to decrease these differences in dielectric constants.

Small ferrite rods can be used within in the coil to improve the quality factor of the coils for better inductive coupling. Innovation in antenna technology includes the use of metal stents as more than structural elements, but also as built-in antennas (*i.e.*, “stentenna”) [31,114]. A “pseudo” normal mode antenna has been developed that has a unique circular polarization profile that decouples the foveation related effects of poor power transfer based on device position [29].

Alternative forms of energy harvesting have been developed that allow the use of piezoelectrics (PZTs) to power some implantable pressure sensors [119]. An acoustic signal generated by an external speaker was transmitted through the body to an implanted piezoelectric cantilever connected to a pressure sensor within the bladder [45]. The low frequency of the acoustic signal allowed for transduction through the body to the PZT with minimal signal loss, and no concerns of orientation or alignment related effects were observed as the acoustic wave field was large enough from the speaker to fully contain the PZT. This particular device decouples the dependence on alignment and separation observed in RF-based telemetry devices and reduces the complexity of the implanted system by removing the need for sophisticated onboard receiver circuitry. A similar technique is used for the Remon ImPressure device, where acoustic waves are used to both transmit power and data to the implanted sensor. For IOP measurements, one technology implanted within the eye takes advantage of a thin-film lithium ion battery and solar cell hybrid [120] that absorbs light entering the eye.

### 4.3. Drift Compensation Strategies

Significant advances in biocompatible fabrication and telemetry have been detailed and discussed above, however drift remains as significant challenge. Sources of drift can be sorted to two categories: those that affect baseline and those that affect sensitivity. The toolbox of techniques to combat drift is ever expanding, as these methods are borrowed and shared amongst all types of transducers.

A common method to remove drift in the baseline measurement involves the comparison of sensor signal to that of a controlled reference. For gauge membrane-based pressure sensors, the effect of the environment on the mechanical properties of the sensing membrane can be the source of baseline drift that can compromise the long-term stability and performance of the implanted device [89,115]. The introduction of a capacitor that is unresponsive to changes in pressure and implanted alongside the sensing element can be used as a reference in a differential signal circuit that subtracts out common effects of drift. Averaging the output from several sensors in an array is a variation of this concept for drift compensation, with possible benefits of extended range and sensitivity. As discussed previously, atmospheric pressure has been used as the baseline reference pressure for many sensing modalities [72,77]. However, several efforts have been made to replace this gauge measurement with a differential type that makes use of another sensor placed at a nearby low-pressure location (e.g., difference in pressure inside *vs.* outside a bladder wall). Benefits of this type of measurement have been adopted with moderate success in improving baseline drift [14].

The addition of temperature data may assist the calibration of sensor response in the *in vivo* environment. Efforts to improve the chronic performance of these sensors make use of an on-board temperature compensation circuit [42,108,121] or external signal processing circuitry [89,118]. Appropriate material selection for certain sensing modalities such as SAW type transducers allow for the use of temperature-insensitive quartz, which circumvents the need for compensation [121,122].

Drift in sensitivity for the commonly used capacitive pressure sensor is primarily caused by the accumulation of biological material on the surface of the device, affecting the mechanical properties of membrane. Removal of the biofouling material is challenging without detrimental effects to the surrounding tissue, and many strategies focus on mechanical approaches rather than chemical [123]. The addition of microstructures to mechanically disturb and break up the cellular matrix have been proposed and developed for other *in vivo* applications may be applied to implantable pressure transducers [124]. Ultrasound has also been used to clear biofilms [123], and may be possible to realize on a SAW or piezoelectric membrane transducer by simply driving the membrane to move at ultrasonic frequencies. Another strategy involves the use of aeration and bubbles to dislodge attached cellular material [125], which can be implemented by adding electrodes on the surface of the implant to perform low-power electrolysis. Long-term water absorption can also play a significant role in affecting mechanical response. Coatings to prevent biofouling and mitigate immune response would also serve to improve drift in sensitivity by shielding the force-transducing membrane from the effects of the body. The development of alternate transduction methods should seek to maintain sensitivity despite the presence of biofouling.

## 5. Prospective Trends

Beyond the development of implantable pressure sensors within the aforementioned areas, trends for chronically implanted pressure sensors center on the use of novel biocompatible electronics and alternative power sources. Briefly, there is considerable development of and vision for biocompatible active electronics, that is organic transistors [86,126] and polymer-based MEMS (*i.e.*, Parylene C) [61,127]. These types of devices can remove the dependence on bulk silicon for the construction of micromachined devices to create implantable sensor and circuit systems that have improved integration with the body once implanted. Taking this concept one step further, the development of biodegradable sensors and batteries [128,129] may enable implantable sensors that can have a limited life-time (currently on the order of days), and then be completely absorbed by the body on demand to remove issues pertaining to a secondary surgery to remove the device following the end of a monitoring time frame or during the event of failure.

The development of alternative power sources outside of RF inductive powering is another area of improvement for implantable pressure sensors. Energy harvesting and power-MEMS is a large and growing area within the MEMS field, with a large portfolio of novel biocompatible devices for use in implantable systems. Movement away from RF inductive powering towards energy harvesting techniques *in vivo* can sever the tether to external units or coils to create a seamlessly integrated implant system. Along these lines, using these energy harvesting devices to power implanted body sensor networks [83,130] and corresponding transmission of the sensed data to the cloud, can greatly improve patient monitoring in the form of wireless health.

Lastly, the field is dominated by the use of membrane-based capacitive or piezoresistive sensing systems for use in implantable pressure systems. Novel sensing mechanisms looking beyond the dependence on the mechanical deflection of a membrane can be insensitive to mechanical fatigue and material stiffness changes, and potentially reduce issues related to drift.

## 6. Conclusions

From the onset of the development of implantable micromachined pressure sensors, the historical challenges of biocompatibility, efficient telemetry, and drift management have persisted. This review highlighted current strategies to improve implantable pressure sensing technology, largely focusing on biocompatible encapsulation along with drug coatings and the development of smart active circuitry to improve telemetric coupling and eliminate drift. The large drift observed in many of the developed sensors may overshadow the improvements gained in novel tissue integration strategies and power/data transmission technology. Further work is needed to address drift management to improve the long-term performance and gain widespread clinical acceptance of these monitoring tools.

## Figures and Tables

**Table 1.** Table of relevant parameters in the development of pressure sensors for implantable applications.

**Application**	**Pressure Range (mmHg) [Ref.]**	**Pressure Resolution (mmHg) [Ref.]**	**Frequency Bandwidth (Hz) [Ref.]**	**Packaging Considerations**
ICP	−10 to 50 [66]	1 [74]	0–30 [82]	Shunt integration, catheter
IOP	12–22, >22 abnormal [65]	2 [94,95]	0–30 [85]	Needle delivery, contact lens
Blood Pressure	50–180 [32]	1 [32]	0–200 [32]	Catheter, stents
Bladder Pressure	10–70, ∼150 during voiding [63]	1 [96]	3–5 [23,83]	Intra-urethral Catheter
Intra-abdominal Pressure	0.2–16.2 [62]	--	0–15 ([84])	--
